# Inbound Call Centers and Emotional Dissonance in the Job Demands – Resources Model

**DOI:** 10.3389/fpsyg.2016.01133

**Published:** 2016-07-28

**Authors:** Monica Molino, Federica Emanuel, Margherita Zito, Chiara Ghislieri, Lara Colombo, Claudio G. Cortese

**Affiliations:** Work and Organizational Psychology, Department of Psychology, University of TurinTurin, Italy

**Keywords:** call center work, job demands-resources model, emotional labor, emotional dissonance, customer verbal aggression

## Abstract

**Background:** Emotional labor, defined as the process of regulating feelings and expressions as part of the work role, is a major characteristic in call centers. In particular, interacting with customers, agents are required to show certain emotions that are considered acceptable by the organization, even though these emotions may be different from their true feelings. This kind of experience is defined as emotional dissonance and represents a feature of the job especially for call center inbound activities.

**Aim:** The present study was aimed at investigating whether emotional dissonance mediates the relationship between job demands (workload and customer verbal aggression) and job resources (supervisor support, colleague support, and job autonomy) on the one hand, and, on the other, affective discomfort, using the job demands-resources model as a framework. The study also observed differences between two different types of inbound activities: customer assistance service (CA) and information service.

**Method:** The study involved agents of an Italian Telecommunication Company, 352 of whom worked in the CA and 179 in the information service. The hypothesized model was tested across the two groups through multi-group structural equation modeling.

**Results:** Analyses showed that CA agents experience greater customer verbal aggression and emotional dissonance than information service agents. Results also showed, only for the CA group, a full mediation of emotional dissonance between workload and affective discomfort, and a partial mediation of customer verbal aggression and job autonomy, and affective discomfort.

**Conclusion:** This study’s findings contributed both to the emotional labor literature, investigating the mediational role of emotional dissonance in the job demands-resources model, and to call center literature, considering differences between two specific kinds of inbound activities. Suggestions for organizations and practitioners emerged in order to identify practical implications useful both to support employees in coping with emotional labor and to promote well-being in inbound call centers. In detail, results showed the need to improve training programs in order to enhance employees’ emotion regulation skills, and to introduce human resource practices aimed at clarifying emotional requirements of the job.

## Introduction

Call center organizations have rapidly increased in the last few decades, attracting considerable attention from different fields including *Work and Organizational Psychology* (Lewig and Dollard, 2003; De Cuyper et al., 2014). The working conditions that can affect call center agents performance and well-being have received particular attention, owing to their influence on organizational success in terms of profit, customer satisfaction and lower costs of absenteeism and turnover (Das, 2012; Rod and Ashill, 2013; De Cuyper et al., 2014). During the last few years, call center operations management has become more focused on staff empowerment and less on a traditional production-line orientation (Gilmore, 2001).

Literature highlighted that call center agents often suffer from burnout and emotional exhaustion (Ashill and Rod, 2011; Choi et al., 2012), and reported emotional dissonance (the discrepancy between expressed and felt emotions, Zapf et al., 1999) as the principal strain phenomenon in call center work (Zapf et al., 1999; Holman et al., 2002; Grebner et al., 2003; Holman, 2003; Lewig and Dollard, 2003; Zapf et al., 2003; Grandey et al., 2004; Ghislieri et al., 2012; Emanuel et al., 2014b). Indeed, in this kind of work there are strong requirements to suppress negative emotions, caused by unfriendly or angry customers, but also by repetitive job activities, cognitive demands, increased time pressure, workload, decreased autonomy and performance monitoring (Holman, 2003; Grandey et al., 2004; Wegge et al., 2006a; Andela et al., 2015).

The present study investigated the role of emotional dissonance within the job demands-resources (JD-R) model (Bakker et al., 2003; Bakker and Demerouti, 2007, 2014) in a sample of call center agents. In particular, the aim of the study was to explore how two job demands (workload and customer verbal aggression) and three job resources (supervisor support, colleague support and job autonomy), typical of the call center context, are related to affective discomfort, a well-being dimension (Warr, 1990), and whether these relationships are mediated by emotional dissonance.

### Call Center Work

Today, many different kinds of companies, in the developed information economies, use call centers as a core way to produce and deliver to the public and customers information services (Russell, 2008). Call centers can be defined as work environments where service agents interact with customers primarily over the phone, or via other communication channels, with the support of computer systems (Van Jaarsveld and Poster, 2013). Since their appearance in the early 1990s, call centers have become an important part of the business world, serving as primary a customer-facing channel for companies with decreased costs of both information technologies and non-specialized personnel, and expectations of high service quality (Grebner et al., 2003; Aksin et al., 2007).

Call centers are generally characterized by structural divisions of labor and extensive use of technology designed to both maximize efficiency and limit worker autonomy and control (task control, timing control and participation) (Knights and McCabe, 1998; Isic et al., 1999; Callaghan and Thompson, 2001). Moreover, there is low complexity and low variability, because the activity consists of routine interactions with customers, controlled mostly by automatic call distribution systems (Holman, 2003). Finally, performance monitoring is a pervasive practice in most call centers, where electronic systems supervise agents controlling quantitative indicators (numbers and length of calls, type of calls taken). Furthermore, the quality of conversations (content, style, adherence to policies) is assessed by recording them and/or listening to them (Holman et al., 2002; Aksin et al., 2007; Moradi et al., 2014).

For these reasons, call center work is demanding, repetitive and often stressful, which can lead to high levels of turnover and absenteeism, and the inability to meet quantitative targets (Taylor and Bain, 1999; Lewig and Dollard, 2003; Workman and Bommer, 2004).

Among the different types of call center activities, previous studies identified that inbound and outbound call center agents perceive stress differently (Zapf et al., 2003; Wegge et al., 2006b; Lewin and Sager, 2007; Lin et al., 2010; Rod and Ashill, 2013). Inbound work is generally focused on helping customers who contact the call center agent, whereas the primary activities of the outbound agent are selling and providing telemarketing with the support of standardized scripts (Lewin and Sager, 2007; Rod and Ashill, 2013). Therefore, inbound call center agents often have to deal with complaints, inquiries and verbal aggression from customers, experiencing greater emotional labor (Aksin et al., 2007; Rod and Ashill, 2013). They are asked to be more customer-oriented and to show abilities such as remaining calm, actively listening, being patient and empathic (Lloyd and Payne, 2009). Moreover, inbound work typically relates to more complex and varied calls than outbound work (Rod and Ashill, 2013).

This study focused on inbound call center work and investigated the differences between two specific kinds of inbound activities, contributing to literature that generally considers only inbound/outbound differences. The first activity considered was the customer assistance service (CA) and consisted in receiving calls from customers who needed to solve some specific technical problems and/or make a complaint; the second was the information service (INFO), aimed at providing phone numbers that customers required.

### Emotional Labor

According to Hochschild (1983), emotional labor can be defined as the process of regulating feelings and expressions as part of the work role (Grandey, 2000). Emotions play an important function in the relationship between employees and customers: companies and managers highlight the importance of this relationship and employees are encouraged formally (or informally) by their organizations to display emotions that conform to certain organizational norms or standards (Zapf et al., 1999; Zapf and Holz, 2006). Thus, expressing appropriate emotions during face-to-face or voice-to-voice interactions has become a job demand for many employees, who are not only required to complete their tasks, but also to conform with specific display rules defined by the corporate culture of an organization (Hochschild, 1983; Grandey, 2000; Schaubroeck and Jones, 2000; Diefendorff and Richard, 2003). Customer service employees are typically encouraged to display a cheerful, friendly manner and behavior while interacting with clients and to express certain emotions (Heuven and Bakker, 2003; Bakker and Heuven, 2006; Humphrey et al., 2015): for example, cabin attendants are expected to display courtesy, police officers firmness, nurses compassion, call center agents willingness.

Therefore, service occupations are considered emotionally demanding for the workers because they must also express certain emotions that may not be felt or may even be opposed to those internally perceived in the situation. Emotional dissonance arises when employees’ expressed emotions are considered acceptable by the organization, but do not represent the true feelings of the individual (Rafaeli and Sutton, 1987; Zapf, 2002; Hülsheger and Schewe, 2011; Grandey and Gabriel, 2015).

In call center work, even though there is no direct face-to-face contact with customers, there are typically strong demands to be friendly with them (Zapf et al., 2003), as type of customer service activities. Moreover, the performance of agents is often monitored by the organization (e.g., test calls or recording calls) and nonconformities from emotional norms can be easily detected (Holman, 2003). In many cases, customers call with problems and call center agents frequently interact with difficult and aggressive people during the workday (Deery et al., 2002; Totterdell and Holman, 2003; Grandey et al., 2004). In particular, customer verbal aggression is descripted as customers’ intentions to damage employees intentionally through words, voice and tone or demeanor such as bad language, shouting and sarcasm (Harris and Reynolds, 2003; Dormann and Zapf, 2004; Grandey et al., 2004). Several studies show that this can undermine employee compliance to regulate emotions: employees, who feel mistreated by customers in both laboratory and field research, seem to force themselves to manage their emotions (Grandey et al., 2004; Rupp and Spencer, 2006; Rohrmann et al., 2011).

Hochschild (1983) was the first scholar to describe the possible negative consequences of emotional labor, for both individuals and organizations. Emotional labor may have potential positive consequences such as the facilitation of interpersonal encounters with customers, task effectiveness, increased service quality or higher income for service providers (Grandey and Gabriel, 2015). However, several scholars have clarified that the regulation of emotions may be especially stressful and detrimental to health: consistent relations have been found between emotional dissonance and burnout complaints across different human service professions (Hülsheger and Schewe, 2011; Kammeyer-Mueller et al., 2013; Kenworthy et al., 2014). Emotional dissonance can have negative consequences for employees. Some scholars underline a positive association with psychological strain, emotional exhaustion, psychosomatic complaints, work-family conflict and lower job satisfaction (e.g., Zapf et al., 1999, 2001; Schaubroeck and Jones, 2000; Zapf, 2002; Grebner et al., 2003; Heuven and Bakker, 2003; Totterdell and Holman, 2003; Wilk and Moynihan, 2005; Cheung and Cheung, 2013; Kenworthy et al., 2014; Scheibe et al., 2015).

Referring to customer relations, Dormann and Zapf (2004) found in three samples of service workers that verbal customer aggression is a strong stressor that is positively correlated with burnout and emotional dissonance. Verbally aggressive customers are a source of strain in the work of call center agents (e.g., Grandey et al., 2004) and the customers’ verbal aggressiveness is an antecedent of emotional dissonance (Wegge et al., 2010). In fact, aggressive customers express and, in turn, foster in agents emotions that employees cannot show according to common emotional rules in call centers (Grandey et al., 2002; Grandey et al., 2004). Therefore, studies in different service jobs found detrimental effects of negative customer behavior on service providers’ well-being (e.g., Dormann and Zapf, 2004; Rupp and Spencer, 2006; Wegge et al., 2007; Wang et al., 2011; Molino et al., 2015).

In this study, a difference was expected between the two types of inbound call center activities in both customer verbal aggression and emotional dissonance. In the CA service, agents have to solve specific and complex technical problems that customers meet with, whereas INFO service agents provide phone numbers that customers require. Customers who call the CA service could be particularly angry and aggressive because of the waste of time and the disappointment about the product or service. Therefore, the first study hypothesis was:

*Hypothesis 1:* (a) CA service agents perceive higher levels of customer verbal aggression than INFO service agents, and (b) CA service agents perceive higher levels of emotional dissonance than INFO service agents.

### JD-R Model and Emotional Dissonance in Call Centers

The JD-R theory (Bakker and Demerouti, 2014) refers to a heuristic model able to specify how two different sets of working conditions may produce both health impairment and motivation (Bakker and Demerouti, 2007). Flexibility of the model permits the theory to be applied to all work environments and occupations, identifying specific job demands and job resources. “Job demands refer to those physical, psychological, social, or organizational aspects of the job that require sustained physical and/or psychological (cognitive and emotional) effort or skills and are therefore associated with certain physiological and/or psychological costs” (Bakker and Demerouti, 2007, p. 312). Job demands are not negative by definition; they become job stressors when meeting those demands requires high effort from which the person cannot adequately recover (Meijman and Mulder, 1998). This study considered two job demands that were identified in call center work. The first one is workload, a general demand investigated in many studies that used the JD-R model (Bakker and Demerouti, 2007; Schaufeli and Taris, 2014); it represents the amount of tasks and activities agents have to manage quickly, handling calls as fast as possible (Wegge et al., 2007). The second one is verbal aggression from unfriendly, rude and/or unsatisfied customers, shown through shouting at service agents and using negative verbal expressions (Dormann and Zapf, 2004; Grandey et al., 2004); customer verbal aggression is a specific demand in inbound call center work.

Job resources represent the second set of job characteristics and “refer to those physical, psychological, social, or organizational aspects of the job that are either/or: functional in achieving work goals; reduce job demands and the associated physiological and psychological costs; stimulate personal growth, learning, and development” (Bakker and Demerouti, 2007, p. 312). Job resources considered in this study were general resources explored in the JD-R model (Bakker and Demerouti, 2007; Schaufeli and Taris, 2014): supervisor support, which indicated the existence of positive and supportive relations between supervisors and agents (Garcia and Archer, 2012; Jansen and Callaghan, 2014); colleague support, which refers to the presence of a collaborative environment among call center agents (Aksin et al., 2007; Garcia et al., 2014); job autonomy, which represents the degree of control over one’s own tasks and behavior at work (Morris and Feldman, 1996).

High levels of job-related stressors and a lack of job resources may negatively affect employees’ well-being (Demerouti et al., 2001). This study examined their relationship with affective discomfort, which refers to the intensity of emotions experienced at work: specifically, high levels of negative emotions are associated with low levels of well-being (Warr, 1990; Van Katwyk et al., 2000; Quaglino et al., 2010; Biggio and Cortese, 2013; Emanuel et al., 2014a).

Moreover, the study examined the mediational role of emotional dissonance in the JD-R model. Several studies have focused on emotional dissonance as mediator in the relationship between job characteristics and employees’ well-being (Bakker and Heuven, 2006; Cheung and Tang, 2007; Karatepe and Choubtarash, 2014; Andela et al., 2015), but few of them referred to call center work. For example, Cheung and Tang (2010), in their study among Chinese call center and retail-shop employees, showed that work characteristics, as manifested by perceived display rules, perceived performance monitoring and perceived service culture, positively influenced strain only through emotional dissonance. Lewig and Dollard (2003), in a study conducted on inbound and outbound call center agents, found that emotional dissonance fully mediated the relationship between emotional demands, expressed by the requirement to display positive emotions, and emotional exhaustion. Our study explored the role of job demands, job resources and emotional dissonance in relation to affective discomfort, a well-being dimension (Warr, 1990); this is important and useful for literature and organizations because our theoretical model was tested in an ever-growing occupational sector in Italy, in which a great many people are employed (Istat, 2014^1^). Furthermore, few studies about call center work in our country have investigated these aspects and the differences among different inbound activities.

The present study considers emotional dissonance as a mediator between job demands and job resources on the one hand, and affective discomfort on the other one. With regard to job demands (workload and customer verbal aggression), they may generate negative emotions that agents cannot show, increasing the experience of emotional dissonance (Hülsheger and Schewe, 2011; Kenworthy et al., 2014; Andela et al., 2015).

Among job resources, supervisor and colleague support may play an important role since they foster a positive working environment in which it is easier for employees to feel positive emotions (Totterdell and Holman, 2003). In customer service work, where the expression of positive emotions is expected, less emotional labor is necessary if the interpersonal relationships are positive and supportive, and positive emotions are genuinely felt (Grandey, 2000). Moreover, support and opportunities to learn from each other may provide agents with those tools and indications helpful to deal with difficult working situations (Moradi et al., 2014), decreasing the likelihood to feel negative emotions. For these reasons, supervisor and colleague support might have a negative relationship with the experience of emotional dissonance.

As for job autonomy, scholars showed that this resource is negatively related to emotional dissonance (Morris and Feldman, 1996). Having autonomy means that agents have more chances to decide how to handle customer calls by thus adapting their answers and behavior to the specificities of both the situation and the customer. In this way, they have more control over the situation, decreasing the likelihood to feel unpleasant emotions that cannot be expressed and, in turn, emotional dissonance perceived.

**FIGURE 1:**
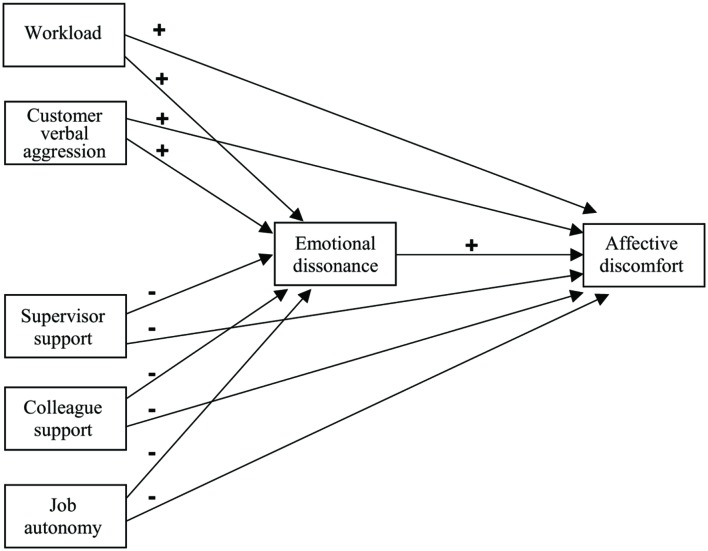
**The theoretical model**.

In conclusion, the aim of the study was to investigate a conceptual model in which two job demands (workload and customer verbal aggression) and three job resources (supervisor support, colleague support and job autonomy) were directly and indirectly, through the mediation of emotional dissonance, related to affective discomfort in inbound call center work. More specifically, the hypotheses were (see **Figure 1**):

*Hypothesis 2:* (a) job demands are positively associated with affective discomfort, and (b) job resources are negatively associated with affective discomfort.
*Hypothesis 3:* (a) job demands are positively associated with emotional dissonance, and (b) job resources are negatively associated with emotional dissonance.
*Hypothesis 4:* emotional dissonance is positively related to affective discomfort, thus playing a mediating role between job demands and resources on the one hand, and affective discomfort on the other.

Since different categories of tasks can generate different emotional and psychological dynamics, and thus different management challenges (Wallace et al., 2000; Jansen and Callaghan, 2014), the hypotheses were tested across the two types of inbound call center activities: the first was the CA service, where customers receive support for specific requests regarding technical problems; the second was the INFO service, which provides customers with phone numbers they need.

## Materials and Methods

### Ethical Statement

The study was conducted in line with the Helsinki Declaration (World Medical Association, 2001), as well as the data protection regulation of Italy. The research project was shared with the trade unions and approved by the Company Board of Directors. Since there was no medical treatment or other procedures that could cause psychological or social discomfort to participants, additional ethical approval was not required. An agreement between the Company and Turin University’s Department of Psychology was signed in order to ensure anonymity and confidentiality in collecting, analyzing and publishing data. Participation in the research was voluntary, without receiving any reward.

### Samples and Procedures

The study was carried out among a national sample of call center agents from an important Italian Telecommunication Company, which provides different ICT services with branches located throughout the country. The aim of the study was explained by sending an e-mail from management and a communication published in the intranet magazine. Anonymity, confidentiality of the data and the voluntary nature of participation in the study were emphasized. A total of 531 call center agents (41.36% of employees involved) filled out the on-line self-report questionnaire.

Participants were from two different kinds of call center activities: 352 of them (66%) worked in the CA, and 179 (34%) worked in the INFO, all of whom worked with an open-ended contract.

The CA sample included 183 females (52%) and 169 males (48%). Their average age was 43.67 years (*SD* = 6.42; min = 25; max = 59). Mean organizational tenure was 19.57 years (*SD* = 7.00; min = 0; max = 37). Most of the participants (63%) worked on a full-time basis. A high percentage of participants (79%) were high school graduates.

The INFO sample included 118 females (66%) and 61 males (34%). Their average age was 38.07 years (*SD* = 7.06; min = 24; max = 56). Mean organizational tenure was 13.81 years (*SD* = 6.95; min = 2; max = 34). Most of the participants (87%) worked on a full-time basis. A high percentage of participants (83%) were high school graduates.

### Measures

*Affective discomfort* was assessed with six items of scale Warr’s, (1990). All items were scored on a 6-point scale, ranging from 1 = *never* to 6 = *all of the time*. Respondents were asked, thinking of the preceding few weeks, how much of the time their job had made them feel, e.g., *“depressed”* or *“gloomy”*. Cronbach’s alpha for the scale in this study was 0.88.

*Emotional dissonance* was assessed with 4 items developed by Zapf et al. (1999). All items were scored on a 6-point scale, ranging from 1 = *never* to 6 = *always*. Respondents were asked, e.g., how often during their work, they had to *“Display emotions which do not correspond to inner feelings”*. Cronbach’s alpha was 0.90.

*Workload* was assessed with six items developed by Karasek and Theorell (1990). All items were scored on a 4-point scale, ranging from 1 = *disagree* to 4 = *agree*. An example item is: “*My job requires working very fast*”. Cronbach’s alpha was 0.82. *Customer verbal aggression* was assessed with 4 items by Dormann and Zapf (2004). All items were scored on a 6-point scale, ranging from 1 = *strongly disagree* to 6 = *strongly agree*. An example item is *“Customers personally attack us verbally”*. Cronbach’s alpha was 0.89.

*Supervisor support* was assessed with 4 items developed by Caplan et al. (1975). All items were scored on a 6-point scale, ranging from 1 = *disagree* to 4 = *agree*. An example item is *“How much was your supervisor willing to listen to your personal problems?”*. Cronbach’s alpha was 0.93. *Colleague support* was assessed with four items (Caplan et al., 1975). All items were scored on a 6-point scale, ranging from 1 = *disagree* to 4 = *agree*. An example item is *“How much was your colleague willing to listen to your personal problems?”*. Cronbach’s alpha was 0.91. *Job autonomy* was assessed with seven items developed by Karasek and Theorell (1990). All items were scored on a 4-point scale, ranging from 1 = *none* to 4 = *a lot*. An example item is *“I can determine the way in which I work”*. Cronbach’s alpha was 0.86.

### Data Analysis

First, descriptive data analysis was carried out in each sample separately (CA and INFO), using the statistics software SPSS 22. Pearson correlations were used to examine the interrelationships between variables. Cronbach’s alpha coefficient was calculated to test the reliability of each scale. Differences in the means of some variables between the two call center services considered were examined by using the analysis of variance (*t*-test for independent samples).

The multi-group structural equation model (SEM) was performed using Mplus 7 (Muthén and Muthén, 1998–2012) in order to assess differences across both samples in the hypothesized model. By running a multi-group model simultaneously for the CA service and INFO service, we tested whether path coefficients differed across the two groups.

The method of estimation was maximum likelihood (ML). According to the literature (Bollen and Long, 1993), the model was assessed by several goodness-of-fit criteria: the χ^2^ goodness-of-fit statistic; the Root Mean Square Error of Approximation (RMSEA); the Comparative Fit Index (CFI); the Tucker Lewis Index (TLI); and the Standardized Root Mean Square Residual (SRMR). Non-significant values of χ^2^ indicate that the hypothesized model fits the data. Values of RMSEA smaller than 0.05 indicate a good fit, values smaller than 0.08 indicate an acceptable fit and values greater than 1 should lead to model rejection. CFI and TLI values greater than 0.95 indicate a good fit. The SRMR has a range from 0 to 1, with a cut-off criterion of 0.08, with higher values indicating poorer fit to the empirical data, and values lower than 0.05 indicating an excellent fit. Finally, bootstrapping was used to test the significance of the mediation hypotheses. The procedure extracted, from the original sample, 2,000 bootstrap samples of the same size as the original one and calculated all direct and indirect parameters of the model (Shrout and Bolger, 2002). When the confidence interval does not include zero it means that there is a significant mediation. The bootstrapping was preferred to other procedures since it was considered a powerful test and was suggested as the best option to test mediation and indirect effects (Shrout and Bolger, 2002).

## Results

**Table 1** shows the means, standard deviations, correlations among the study variables and internal consistency of each scale, separately for CA and INFO samples. All α values meet the criterion of 0.70 (Nunnally and Bernstein, 1994) as they ranged between 0.82 and 0.94.

**Table 1:** Item means, item standard deviation, Cronbach’s alphas, and correlations among the study variables for CA (*n* = 352) and INFO (*n* = 179).

	CA		INFO								
	*M*	*SD*	*M*	*SD*	*1*	*2*	*3*	*4*	*5*	*6*	*7*
1. Affective discomfort	2.98	1.12	3.13	1.31	*0.86/0.90*	0.21^∗∗^	0.28^∗∗^	0.39^∗∗^	-0.32^∗∗^	-0.13	-0.28^∗∗^
2. Emotional dissonance	3.94	1.33	3.58	1.35	0.42^∗∗^	*0.90/0.91*	0.31^∗∗^	0.31^∗∗^	-0.12	-0.11	-0.25^∗∗^
3. Workload	2.93	0.61	2.82	0.66	0.22^∗∗^	0.41^∗∗^	*0.82/0.83*	0.16^∗^	-0.14	-0.04	-0.16^∗^
4. Customer verbal aggression	4.23	1.20	3.63	1.36	0.25^∗∗^	0.31^∗∗^	0.27^∗∗^	*0.88/0.90*	-0.24^∗∗^	-0.06	-0.33^∗∗^
5. Supervisor support	4.70	1.31	4.25	1.33	-0.29^∗∗^	-0.16^∗∗^	-0.08	-0.05	*0.94/0.91*	0.29^∗∗^	0.27^∗∗^
6. Colleague support	4.75	1.16	4.63	1.01	-0.14^∗∗^	-0.06	0.10	-0.01	0.46^∗∗^	*0.92/0.88*	0.12
7. Job autonomy	2.07	0.64	2.52	0.64	-0.35^∗∗^	-0.37^∗∗^	-0.39^∗∗^	-0.20^∗∗^	0.22^∗∗^	0.05	*0.86/0.84*

*Correlations for the CA are below the diagonal; correlations for the INFO are above the diagonal. Italic values on the diagonal are Cronbach’s α for CA/INFO sample. ^∗^*p* < 0.05; ^∗∗^*p* < 0.01.*

All the significant correlations between the variables were in the expected directions. Affective discomfort was positively correlated with job demands (workload and customer verbal aggression) and negatively associated with two job resources (supervisor support and job autonomy), across samples. In both samples, affective discomfort was positively associated with customer verbal aggression (CA: *r* = 0.25, *p* < 0.01; INFO: *r* = 0.39, *p* < 0.01) and workload (CA: *r* = 0.22, *p* < 0.01; INFO: *r* = 0.28, *p* < 0.01), negatively associated with job autonomy (CA: *r* = -0.35, *p* < 0.01; INFO: *r* = -0.28, *p* < 0.01) and supervisor support (CA: *r* = -0.29, *p* < 0.01; INFO: *r* = -0.32, *p* < 0.01). Only in the CA sample, affective discomfort was positively associated with colleague support (CA: *r* = -0.14, *p* < 0.01). Emotional dissonance related positively to affective discomfort across both samples but with a stronger relationship in the CA one (CA: *r* = 0.42, *p* < 0.01; INFO: *r* = 0.21, *p* < 0.01). Furthermore, emotional dissonance was positively associated with job demands, and negatively associated with job autonomy, across samples. Among job demands, workload (CA: *r* = 0.41, *p* < 0.01; INFO: *r* = 0.28, *p* < 0.01) and customer verbal aggression (CA: *r* = 0.31, *p* < 0.01; INFO: *r* = 0.39, *p* < 0.01) showed a significant positive correlation with emotional dissonance, in both samples. Among the other job resources, job autonomy (CA: *r* = -0.37, *p* < 0.01; INFO: *r* = -0.25, *p* < 0.01) showed a significant negative correlation with emotional dissonance, in both samples; supervisor support showed a significant negative correlation with emotional dissonance only in the CA sample (CA: *r* = -0.16, *p* < 0.01) and colleague support was not correlated with emotional dissonance in both samples.

Hypothesis *1a* stated that the CA sample perceived higher levels of customer verbal aggression than the INFO sample. Analysis of variance between the two samples showed a difference in the customer verbal aggression: individuals working in the CA call center perceived more customer verbal aggression (*M* = 4.23, *SD* = 1.20) than individuals working in the INFO call center (*M* = 3.63, *SD* = 1.36) [*t* (321) = 4.99, *p* < 0.01]. Hypothesis *1b* stated that the CA sample perceived higher levels of emotional dissonance than INFO sample. Individuals working in the CA call center experienced more emotional dissonance (*M* = 3.94, *SD* = 1.33) than individuals working in the INFO call center (*M* = 3.58, *SD* = 1.35) [*t* (529) = 2.94, *p* < 0.01]. Hypothesis *1* was therefore fully confirmed. Furthermore, possible differences in the affective discomfort were investigated: no differences between the CA and INFO samples were found for the mean levels of affective discomfort.

The multi-group SEM of the hypothesized model (**Figure 1**) was first evaluated by constraining all the path coefficients to be equal across the two groups. Subsequently, the model was re-tested relaxing the constraints that significantly increased the fit if they were estimated freely across the two groups, consistent with the theory (Bollen, 1989). The final model fitted to the data well: χ^2^ (9, N_CA_ = 352, N_INFO_ = 179) = 10.51, *p* = 0.31, CFI = 0.99, TLI = 0.99, RMSEA = 0.03 (90% CI 0.00, 0.08), SRMR = 0.02. A significant chi-square difference between the two models suggested this final model fitted the data better than the fully constrained model, Δχ^2^ (2) = 6.49; *p* < 0.05 (Satorra and Bentler, 1999).

**FIGURE 2:**
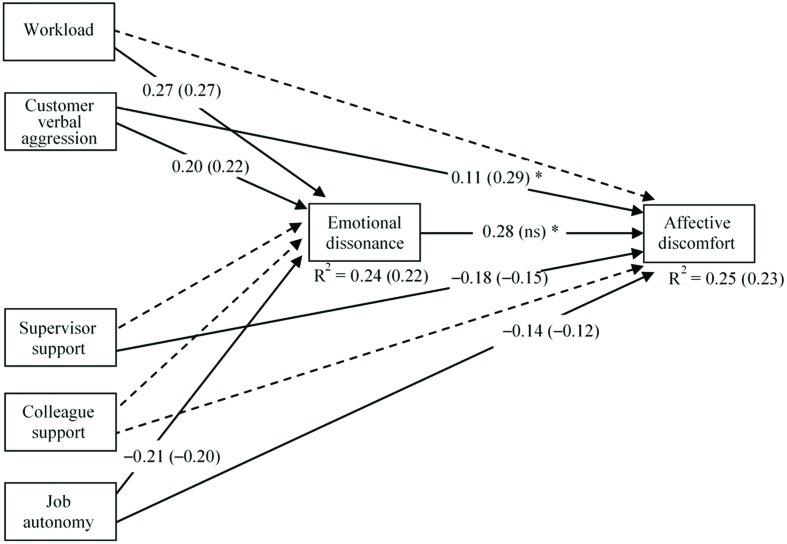
**The final model (standardized path coefficients, *p* < 0.05)**. Results of the multi-group analysis: CA (INFO). Coefficients with asterisk are significantly different. Discontinuous lines indicate non-significant relationships.

**Figure 2** shows standardized parameters derived from the re-specified model. In this model, the differences in standardized parameter estimates of the constrained paths between the groups reflected group specific differences in variances of variables. Hypothesis *2a* stated that job demands are positively associated with affective discomfort. Customer verbal aggression showed a significantly stronger positive relationship with affective discomfort in the INFO sample than the CA sample. Workload did not show a direct relationship with affective discomfort in both samples. With regard to job demands, Hypothesis *2a* was partially confirmed. Hypothesis *2b* stated that job resources were negatively associated with affective discomfort. Supervisor support and, with a weaker loading, job autonomy, were negatively related to affective discomfort, across the two groups. Colleague support did not show direct relationships with affective discomfort across the two samples. With regard to job resources, Hypothesis *2b* was partially confirmed. Hypothesis *3a* stated that job demands were positively associated with emotional dissonance. Workload and customer verbal aggression showed significant positive relationships with emotional dissonance, across the two samples: Hypothesis *3a* was fully confirmed. Hypothesis *3b* stated that job demands were negatively associated with emotional dissonance. Among job resources, only job autonomy had a significant negative relationship with emotional dissonance, in both samples, supervisor support and colleague support did not show significant relationships with emotional dissonance. With regard to job resources, Hypothesis *3b* was partially confirmed.

Hypothesis *4* stated that emotional dissonance was positively related to affective discomfort, playing a meditational role between job demands and resources on the one hand, and affective discomfort on the other. Emotional dissonance showed a significant positive relationship with affective discomfort only in the CA sample. The mediating paths in the CA sample were evaluated using a bootstrapping procedure, **Table 2** presents these results and shows that all the mediated effects, in the CA sample, were statistically significant. Particularly, the bootstrapping procedure confirmed that in the CA sample, emotional dissonance fully mediated the relationship between workload and affective discomfort. Moreover, emotional dissonance was a partial mediator between customer verbal aggression and affective discomfort, and between job autonomy and affective discomfort. In the INFO sample, emotional dissonance did not show a mediational role and, therefore, the bootstrapping procedure was not applied. Therefore, Hypothesis *4* was confirmed only in the CA sample. Variance of dependent variables explained by the models was 25% for affective discomfort and 24% for emotional dissonance in the CA sample; 23% for affective discomfort and 22% for emotional dissonance in the INFO sample.

**Table 2:** Significant indirect effects using bootstrapping (N_CA_ = 352).

Indirect effects	Bootstrap			
	Est.	S.E	*p*	CI 95%
Workload → Emo. Diss. → Aff. Disc.	0.10	0.02	0.000	(0.04, 0.11)
Cust. Verbal Aggr. → Emo. Diss. → Aff. Disc.	0.06	0.02	0.000	(0.03, 0.09)
Job Aut. → Emo. Diss. → Aff. Disc.	-0.06	0.02	0.001	(-0.09, -0.02)

*Emo. Diss., emotional dissonance; Aff. Disc., affective discomfort; Cust. Verbal Aggr., customer verbal aggression; Job Aut., job autonomy; CI, confidence interval. All parameter estimates are presented as standardized coefficients.*

## Discussion

This study examined the role of emotional dissonance in the JD-R model, investigating whether it mediated the relationship between job demands and resources, and affective discomfort, referring to the Italian call center context. As such, the study contributed to the emotional labor literature, focusing on specific antecedents of emotional dissonance and confirming its mediational role. The study also addressed a gap in the call center literature, as it was one of the first that considered differences between two specific kinds of inbound services, despite many that studies focused mainly on inbound/outbound differences (Lin et al., 2010; Rod and Ashill, 2013).

Among the job demands considered, customer verbal aggression showed a direct positive relationship with both emotional dissonance and affective discomfort. As for workload, the relationship with affective discomfort was fully mediated by emotional dissonance. These results confirmed that not only aggression from customers, which generated negative emotions the agent cannot display, was an antecedent of emotional dissonance (Hülsheger and Schewe, 2011), but also the amount and pressure of work could generate negative feelings, which employees cannot express during the job. Managing these opposite emotional experiences might be more difficult when there is a high level of requests and tasks to do. Therefore, in this context, workload was a demand strongly associated with emotional dissonance and, indirectly through the mediation of emotional dissonance, to affective discomfort.

Regarding job resources, supervisor support and job autonomy had a direct negative relationship with affective discomfort in both call center activities, confirming previous studies that demonstrated that a supportive climate, and having autonomy in the job, contributed to employees well-being (Grandey, 2000; Emanuel et al., 2014a). Moreover, the study confirmed a negative relationship between job autonomy and emotional dissonance (Morris and Feldman, 1996). Particularly, results confirmed that employees who perceived more autonomy in the job were able to deal better with emotional dissonance, likely because of greater discretion in choosing how to manage calls with customers and how to deal with difficult situations, preventing negative reactions from the customers and bad feelings. Colleague support did not show any of the expected relationships with emotional dissonance or with affective discomfort. In the two call center contexts, despite colleagues being perceived as supportive, the activities were typically carried out at individual level (Moradi et al., 2014). Moreover, the opportunities to interact with each other and give advices might not have been sufficient and adequate to work as factors able to protect agents from experiencing affective discomfort and helping in dealing with emotional dissonance. Similarly, regarding supervisors, results indicated that their support was not functional to decrease the experience of emotional dissonance.

Finally, the study tested the meditational role of emotional dissonance among job demands and job resources and affective discomfort. First, emotional dissonance was related to affective discomfort only in the case of the CA sample, where agents had to provide technical and specific customer service. In this sample, it fully mediated the relationship between workload and affective discomfort, and partially mediated the relationship between customer verbal aggression and job autonomy on the one hand, and affective discomfort on the other. Call center inbound services aimed at providing support, as in the CA one, were characterized by more aggressive behavior from customers, who are generally angry, frustrated or unsatisfied, and vent their discontent on agents (Dormann and Zapf, 2004; Grandey et al., 2004). Moreover, the activity was particularly difficult and problematic, and requests were rarely predictable (Rod and Ashill, 2013). The specific features of this kind of job might increase the possibility to experience emotions that could not be shown. Consequently, agents in this service perceived more emotional dissonance (Lewig and Dollard, 2003; Zapf et al., 2003). In the case of the INFO sample, emotional dissonance did not have a mediational role between job demands and job resources, and affective discomfort. In fact, emotional dissonance was not related to affective discomfort. The analysis of variance showed that in the INFO service, agents perceived less emotional dissonance, compared with colleagues in the CA service. INFO service agents provided phone numbers that customers required and were less exposed to customer verbal aggression. Therefore, starting from these features of work, emotional dissonance in INFO services seemed to be a less critical variable, which did not relate to affective discomfort.

### Limitations and Future Studies

The present study used a cross-sectional research design that did not permit establishing causality relations between variables (Podsakoff et al., 2012). Further studies should examine the longitudinal effects of emotional dissonance on negative outcomes, such as burnout and exhaustion, thus supporting our hypotheses even more. Additionally, future research will benefit from adding physiological measures of occupational stress (Quaglino et al., 2010) and well-being, such as blood pressure and heart rate (Ilies et al., 2010), and other objective organizational measures, such as the absenteeism rate.

A second limitation is the exclusive use of self-reported questionnaires that can potentially contaminate results, because observed relationships may be artificially inflated because of the respondents’ tendency to answer in a consistent manner. Nevertheless, self-reported data seemed to be the most appropriate approach in our study as it evaluated workers’ subjective perceptions of job demands, job resources, emotional dissonance and affective discomfort.

Another limitation was that data was collected from one organization only, which restricted the generalization of our findings. However, it is important to note that participants were employees from two different call centers of the same organization, with different kinds of inbound activities. Results from a relatively heterogeneous sample of employees supported previous findings that showed that emotional labor and emotional dissonance at work applied to a wide array of occupational contexts (Kenworthy et al., 2014). Replication of the current findings in future studies conducted in various (service and non-service) organizations is essential and important.

Finally, in our study, supervisor support and colleague support did not show the expected relationships with the other variables, although theory and research underlined that social support was a relevant job resource for well-being in emotionally laden jobs (Grandey, 2000; Totterdell and Holman, 2003). Future studies should consider social support as a possible moderator variable (Grandey, 2000; Grandey and Gabriel, 2015) in order to verify its buffer effect in these dynamics.

## Conclusion and Practical Implications

The results of the current study contribute to the existing literature on emotional labor and affective discomfort in service occupations, especially in call center work. Useful implications for both researchers and practitioners emerged, in order to understand better which job demands and job resources, typical for call center work, were more related to well-being and discomfort at work. In addition, the study, which dealt with two inbound services, allowed us to identify not only common implications for two kind of activities but also different implications based on their distinctive features (Wallace et al., 2000; Jansen and Callaghan, 2014).

First, the results were important to understand better the role of job demands and job resources, typical for this occupation. In line with previous studies, the possibilities to promote well-being in these call center services (CA and INFO services) were, for example, improving the control and autonomy of agents (Zapf, 2002; Johnson and Spector, 2007) or promoting a positive and supportive work climate (Garcia and Archer, 2012; Jansen and Callaghan, 2014), and reducing or redistributing the workload (Wegge et al., 2006b).

An important practical path for organizations and management to take would be the development of training programs to enhance employees’ emotion regulation skills in order to cope with customer mistreatment (Grandey et al., 2004; Groth, 2005; Rupp et al., 2008) and to improve emotion regulation strategies. However, generally, training in organizations has not been directly applied to emotional labor, and training is often invested only for managers and leaders, not service workers. Results suggest that it is important to develop training programs for all service workers, as well as call center agents. Training could help employees to understand the negative consequences of emotional labor and to identify what kind of strategy is useful to cope with daily demands, in particular for CA service agents that receive calls from customers who need to solve technical problems and/or make a complaint. In fact, enhancing emotional competency could help CA service workers to handle their emotional work better, reduce stress and increase the level of well-being (Giardini and Frese, 2006; Gabriel et al., 2016; Zito et al., 2016), as shown in nursing employment (McQueen, 2004). Moreover, recent studies underlined the importance to improve emotional intelligence (Boyatzis et al., 2002; Gabriel et al., 2016), emotional self-efficacy (Pugh et al., 2011), and peer-rated emotional competence (Giardini and Frese, 2006) through training programs, to help employees effectively engage in emotional labor. In fact, several scholars showed that emotional competency could reduce emotional demands and sustain well-being at work (Pugh et al., 2011; Gabriel et al., 2016). In this organizational context, training programs about emotional competency could be useful and beneficial for the two inbound services considered in this study (CA and INFO services).

Another practical path for call center companies would be to enhance the presence of human resource practices for emotional labor, which can increase commitment to emotional goals (Gosserand and Diefendorff, 2005; Diefendorff and Croyle, 2008; Gabriel et al., 2016). Companies could also engage training programs for supervisors in order to recognize and support the effort required by emotional labor to call center agents. In fact, it would be important to create an employee-supportive (rather than managerial-controlling) climate (Bono et al., 2007; Nishii et al., 2008). Training programs for supervisors would be particularly useful for the CA service in order to sustain call center agents that perceive more emotional dissonance and customer verbal aggression. Some studies also found that management tactics, such as monitoring and reward, did not make emotional labor more controlled and distressing (Hochschild, 1983). Performance monitoring in call centers did not increase emotional labor and strain if the perceived purpose of monitoring was supportive (Holman et al., 2002), and financial incentives enhanced satisfaction from emotional labor (Grandey et al., 2013). Moreover, socialization could also be used to increase identification with organization goals, which would buffer strain from emotional labor (Schaubroeck and Jones, 2000; Grandey and Gabriel, 2015). Moreover, mentors who provide vocational and psychosocial support and serve as role models, could help ground staff members to manage their emotions, mitigate emotional dissonance and experience lower emotional exhaustion (Karatepe, 2013; Kim et al., 2013), in order to reduce potential undesirable outcomes, such as turnover intentions and absenteeism. Referring to this organizational context, socialization and mentoring programs could be advantageous for the two inbound services, because these actions could increase awareness in relation to job role and work-related activities, and useful for CA and INFO services agents.

Referring to recruitment, it has been suggested that by clarifying the emotional labor requirements during the selection process, individuals may have a well-defined idea of what is expected (Wanous, 1992) and, in this specific organizational context, it might be advantageous for the two inbound services (CA and INFO services). Making emotional requirements explicit during recruitment should create expectations about emotional performance (Rafaeli and Sutton, 1987). However, many organizations lack explicit policies regarding emotional displays and norms to guide customer service behavior, with some referring to them only vaguely in their mission declarations (Zapf, 2002; Constanti and Gibbs, 2005). Many studies (Chen and Lin, 2009; Bartram et al., 2012) showed that when employees are selected to perform emotional labor, burnout is reduced. Other scholars (Callaghan and Thompson, 2002; Grandey and Gabriel, 2015; Gabriel et al., 2016) highlighted that competencies embedded in personality (positive attitude, sense of humor, enthusiasm, extraversion), technical skills (typing, navigation), and communication (energy, fluency, warmth, tone) should be used for selection in emotionally laden jobs. Moreover, scholars have suggested recruiting and selecting individuals whose skills match the emotional display rules for a specific organization and/or role (Glomb et al., 2004; Constanti and Gibbs, 2005).

Finally, the results of this study provided a contribution to current literature on emotional labor and affective discomfort in service occupations, especially in call center work, and identified differences between two different types of inbound activities. In particular, emotional dissonance mediates the relationship between workload and customer verbal aggression and affective discomfort for CA agents. These results suggested the importance of monitoring the experiences of emotional dissonance and emotional labor in call center work and the negative consequences for employees, in order to sustain workers and promote well-being in inbound call centers and in general in service jobs.

## Author Contributions

All authors (MM, FE, MZ, CG, LC, CC) contributed to this work. MM and FE developed and designed the study, wrote the manuscript and received substantial input from co-authors. CG and LC collected the data. CG supervised the research team and contributed to introduction and discussion sections of the manuscript. LC and MZ contributed to methods and data analysis. CC contributed to conclusion and practical implications section of the manuscript. All authors approved the final version of the manuscript for submission.

## Conflict of Interest Statement

The authors declare that the research was conducted in the absence of any commercial or financial relationships that could be construed as a potential conflict of interest.

## Footnotes

1 http://www.istat.it/it/archivio/125372
